# Stability of performance in a hierarchical system during isometric force production and effects of visual feedback

**DOI:** 10.1007/s00221-025-07202-9

**Published:** 2025-11-27

**Authors:** Sayan Deep De, Xiaogang Hu, Muhammad Saad Khan, Mark L. Latash

**Affiliations:** 1 Department of Kinesiology, The Pennsylvania State University, Rec.Hall-268N, University Park, PA 16802 USA; 2 Department of Mechanical Engineering, The Pennsylvania State University, University Park, PA 16802 USA

**Keywords:** Force production, Hierarchical control, Visual feedback, Synergy, Finger

## Abstract

We address the underexplored role of visual feedback in ensuring stability of pressing force produced in isometric conditions by the two hands. We considered the task as a two-level hierarchy (total force, F_TOT_, and hand forces, F_HAND_) and used the framework of the uncontrolled manifold hypothesis to quantify synergies stabilizing F_TOT_ and F_HAND_. Young healthy persons performed steady-state forces with visual feedback and targets for the forces produced by each hand and/or for the total force. We quantified inter-trial and within-a-trial (across time windows) variance components affecting and not affecting F_TOT_ and F_HAND_. Variables with visual feedback, F_TOT_ or F_HAND_, showed force-stabilizing synergies reflected in the structure of variance, while variables without visual feedback failed to show such synergies. The synergies were consistently stronger when the feedback was continuous, compared to when the feedback disappeared when the cursor was inside the target. The results showed a strong dependence between variance that did not affect F_TOT_ (within the corresponding solution space) and variance that affected each F_HAND_ across the feedback conditions. Within-a-trial synergy indices were significantly lower than those in the inter-trial analysis. The data are interpreted as reflecting hierarchical control with synergies at both levels of the hierarchy, those that define F_TOT_ and F_HAND_. These synergies get contributions from inter-trial force sharing variability and sensory feedback-based covariation of forces produced by the elements (fingers or hands) along individual trials. Combining inter-trial and within-a-trial analysis of variance may provide an important toolbox to explore cases of impaired control of action stability.

## Introduction

All everyday human movements involve large sets of elements (e.g., joints, digits, muscles, motor units, etc.) contributing to relatively low-dimensional salient, task-specific performance variables. Bernstein (1947) introduced the concept of *synergy* as a neural organization with two main functions. First, synergies organize elements into a smaller number of groups (addressed with different terms such as modes, factors, or primitives, reviewed in Latash 2020) with the purpose to alleviate the problem of motor redundancy. Second, synergies ensure dynamical stability of the salient performance variables by co-varying the contributions of elements. In this context, stability implies an ability of a system of interest to stay close to an intended state or trajectory following small changes in the system’s intrinsic state and/or external force (according to the Lyapunov definition of stability).

The uncontrolled manifold (UCM) hypothesis (Scholz and Schöner 1999) has become a popular method for analyzing the stability of performance variables produced by such sets of elements (for a recent review, see Latash 2021, 2024, 2025). According to the UCM hypothesis, the central nervous system (CNS) organizes interactions among elements (cf. the principle of abundance, where the components are viewed not as redundant but as essential to maintain stability of performance variables, Gelfand and Latash 1998; Latash 2012) to channel the unavoidable variability in their outputs into a subspace where the magnitude of a salient performance variable remains unchanged. This subspace has been addressed as the uncontrolled manifold (UCM) for that variable. Most common methods of analysis involve quantifying inter-trial variance along the UCM and along the orthogonal to the UCM (ORT) space, V_UCM_ and V_ORT_. An index of a synergy stabilizing the salient performance variable in the space of elemental variables, e.g., ∆V = (V_UCM _− V_ORT_)/(V_UCM_ + V_ORT_), with each of the two variance components quantified per dimension, has been used in several basic and applied studies. Note that ∆V reflects the structure of variance between the UCM and ORT, not its absolute magnitude, and is not synonymous with performance accuracy. According to this definition, ∆V > 0 can be interpreted as the existence of a synergy stabilizing the task-specific performance variable, larger ∆V magnitudes correspond to stronger synergies, while ∆V ≤ 0 implies the lack of such a synergy. In the current study, we use the described theoretical framework to address a number of underexplored and ambiguous issues such as the relations between the V_UCM_ and V_ORT_ in a hierarchical system, the origins of V_UCM_ and their dependence on visual feedback, and the possibility to use single trials to infer stability of performance during accurate force production in isometric conditions.

**Fig. 1:**
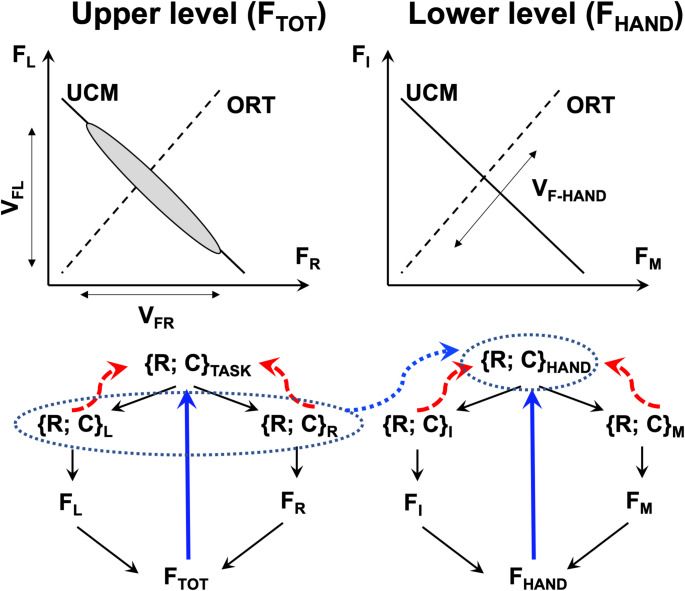
The upper panels show the solution space (uncontrolled manifold, UCM) and orthogonal to the UCM space (ORT) for the task-level output. A force-stabilizing synergy at the Upper level corresponding to stability of total force (F_TOT_) is reflected in the inter-trial data cloud forming an ellipse elongated along the UCM. Note that projections of this ellipse on the hand force axes (F_R_ and F_L_, for the right and left hand) are expected to define variance (V_FR_ and V_FL_, respectively) along the ORT at the Lower level (V_F-HAND_) illustrated in the top, right panel (F_I_ and F_M_ stand for forces by the index and middle fingers). The lower schemes show the assumed control of a two-hand, four-finger force production task. For each effector, two control variables are specified, reciprocal and coactivation, {R; C}. At the Upper level (left), the {R; C} pair for total force, {R; C}_TASK_, are shared between the {R; C} pairs for the right and left hands, {R; C}_R_ and {R; C}_L_. These result in the generation of the hand forces (F_R_ and F_L_ for the right and left hands), which sum-up to the total force, F_TOT_. At the Lower level, the {R; C} pair for each hand, {R; C}_HAND_, is shared between {R; C} pairs for the index and middle fingers ({R; C}_I_ and {R; C}_M_). These result in the generation of the finger force (F_I_ and F_M_ for the index and middle fingers), which sum-up to the hand force, F_HAND_. The blue thick lines show visual feedback (if available). The dashed red curved lines show back-coupling loops within the central nervous system. Note the correspondence between the control pairs, {R; C} at Upper level (left) and Lower level (right) shown with the blue dotted ellipses and line

All everyday movements may be viewed as involving hierarchies of elements, from the whole body to limbs, to digits, to joints, to muscles, and to motor units. One of the unexpected findings was that stabilization of a performance variable produced by a set of elements was in competition with stabilization of the contributions of each of the elements by co-varying the contributions of elements at the next, lower level (Gorniak et al. 2007). For example, stabilizing the total pressing force by two hands at the higher level is in competition with stabilization of the individual hand forces by co-varying the contributions of the individual digits at the lower level. In more general terms, a strong performance-stabilizing synergy at one of the levels suppresses the ability to express a strong synergy at the other level, i.e., one cannot have high ∆V values at both levels. The theoretical analysis corroborated by those original studies suggested that the trade-off between performance-stabilizing synergies at different hierarchical levels resulted from a dependence of V_ORT_ at the lower level on V_UCM_ at the upper level (see the upper panels of Fig. 1). The primary goal of our study has been to test this hypothesis directly (Hypothesis-1) across a variety of visual feedback conditions, which were selected to stabilize (∆V > 0) or not stabilize (∆V ≈ 0) performance at different hierarchical levels. Note that visual feedback plays a crucial role in accurate constant force-production tasks as shown, for example, by studies of force drifts after the visual feedback on the force magnitude has been turned off (Vaillabcourt and Russell 2002; Ambike et al. 2015). Within our experimental design, the upper level of the hierarchy corresponded to sharing total force (F_TOT_) between the two hands, and the lower level corresponded to sharing the force produced by each hand (F_HAND_) between the two involved fingers, index and middle.

The bottom panels of Fig. 1 illustrate the conceptual design of our study within the theory of hierarchical control with pairs of basic commands, reciprocal and coactivation (*R* and *C*), reflecting spatial referent coordinates to the agonist and antagonist muscle groups (reviewed in Latash 2010; Feldman 2015). The *R*-command defines a spatial coordinate where the forces of the opposing muscle groups are balanced, and the *C*-command defines a spatial range where both agonist and antagonist muscles show non-zero activation. During force production in isometric conditions, changes in both *R*-command and *C*-command affect force magnitude, the latter by a change in the apparent stiffness of the effector (De et al. 2025). The control of a two-hand, four-finger force production task involves both within-the-CNS feedback loops (dashed curved arrows, cf. Latash et al. 2005) and also feedback loops from sensory receptors (straight arrows, cf. Martin et al. 2009, 2019), in particular visual feedback signals on the magnitude of each F_HAND_ as well as of F_TOT_.

Our specific experimental design is based on a series of recent studies (Abolins et al. 2025a, b), which used a two-finger force production task with targets for individual finger forces in addition to a target for F_TOT_. Those studies tested and supported the hypothesis that V_UCM_ comprised two distinct components: variability in the sharing (V_UCM,SH_) of F_TOT_ between finger forces, which is seen across trials, and covariation among finger forces (V_UCM,CoV_) based on sensory information, which is seen over time along each of the trials. Our study was designed to test this hypothesis on the two origins of V_UCM_ for a hierarchical system: The task-related F_TOT_ was produced by two hands, while each hand’s force was produced by two fingers per hand. The current study also adds an important new condition when no feedback on F_TOT_ is available, while variations in the magnitude of F_TOT_ (and, therefore, the V_ORT_) are constrained by the explicit targets for the forces produced by each hand (F_HAND_). The schematics in Fig. 1 suggest that, when feedback on both F_HAND_ is available without feedback on F_TOT_, no F_TOT_-stabilizing synergies will be seen (∆V ≈ 0; V_UCM_ ≈ V_ORT_: Hypothesis-2) even though the small targets for F_HAND_ naturally limits V_ORT_ for F_TOT,_ making Hypothesis-2 highly non-trivial and central to our study. Supporting this prediction would underscore the important difference between the stability of a variable and the accuracy of its performance. The latter depends only on V_ORT_ because, by definition, V_UCM_ has no effects on the performance variable. The former is reflected in the relative amounts of V_ORT_ and V_UCM_ (see the earlier definition of ∆V, the synergy index).

The studies of Abolins et al. (2025a, b) used a non-trivial feature of visual feedback: It was available only when the cursor showing the magnitude of a force variable was outside the target and was not seen when the cursor was within the target borders. During the steady-state phases, this effectively led to intermittent visual control of the task (cf. Slifkin et al. 2000; Sosnoff et al. 2005; Hu and Newell 2011; Lafe et al. 2016): Corrections were expected only when the cursor moved out of the target zone. If visual information is available continuously, at all times, corrections of the cursor deviations from the center of the target may be expected without the cursor leaving the target zone, resulting in higher ∆V magnitudes due to smaller V_ORT_ (Hypothesis-3, which may be viewed as a secondary one). This hypothesis was tested using conditions with continuous and off-target visual feedback.

Our last, exploratory, goal was to use inter-sample analysis over single long trials with constant F_TOT_ production instead of the commonly used inter-trial analysis, which requires collecting numerous trials (cf. De Freitas et al. 2018; Piscitelli et al. 2024). Obviously, analysis of single trials cannot reflect the hypothesized V_UCM,SH_ component since V_UCM,SH_, by its definition, reflects differences across trials only. On the other hand, during constant F_TOT_ production with the help of visual feedback on F_TOT_ magnitude, subjects tend to exhibit predominantly negative covariation of individual finger forces, effectively keeping the combined output close to the UCM for F_TOT_ (Ranganathan and Newell 2008a, b; Hu et al. 2011). Hence, single-trial analysis can be used as a source of information on the V_UCM,CoV_ component. Developing a method that allows distinguishing between the two components is potentially important for understanding disorders of motor coordination in clinical populations with significantly reduced V_UCM_ (e.g., Falaki et al. 2017; Jo et al. 2017). To achieve this goal, we tested Hypothesis-4 that, with feedback on F_TOT_, inter-sample (within-a-trial) analysis would show smaller ∆V values for F_TOT_ compared to the inter-trial analysis, although both would be positive. Given the potential practical importance of Hypothesis-4, we consider it as another major hypothesis in the study.

## Methods

### Subjects

Fourteen participants (seven females and seven males) with an age of 23 ± 3 years (mean ± SD) participated in the experiment. Their average mass was 71.6 ± 13.5 kg, and average height was 1.7 ± 0.09 m. The sample size was selected based on earlier studies examining performance-stabilizing synergies using the uncontrolled manifold (UCM) analysis, indicating medium-to-large effect sizes on synergy indices (Park et al. 2012; Solnik et al. 2015; Abolins et al. 2025b). All participants were healthy, without any known neurological or muscular disorders. Participants were right-handed based on their preferential hand usage during daily activities such as drawing and eating. Normal or corrected-to-normal vision was required for participation. The research adhered to the guidelines established by the most recent version of the Declaration of Helsinki and was conducted under a protocol approved by the Penn State Institutional Review Board. All participants provided written informed consent.

### Apparatus

**Fig. 2:**
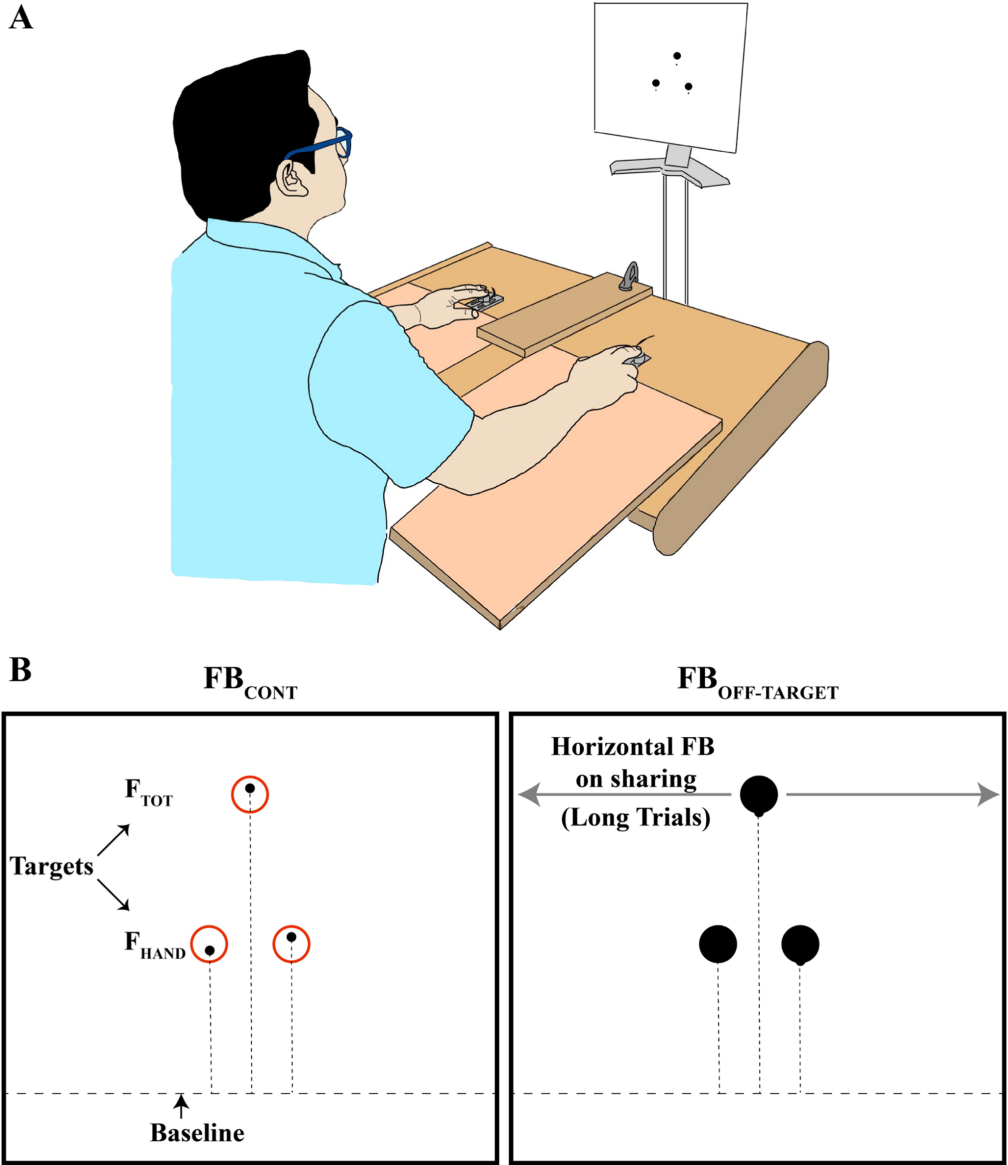
**A** Schematic illustration of the setup. **B** Illustration of the continuous feedback (left) and off-target feedback (right) conditions. Note the three targets in each panel: for total force (F_TOT_) and for the forces produced by the pairs of fingers in the individual hands (F_HAND_)

Participants were seated in a sturdy chair with back support, positioning their forearms comfortably on wooden platforms attached to a rigid table in front of the monitor (Fig. 2A). Four force/torque transducers (Nano-17 sensors, ATI Industrial Automation, Garner, NC, USA) were arranged within rigid frames (each measuring 140 × 90 mm). These sensors were used to measure the vertical forces exerted by the index (I) and middle (M) fingers of each hand. To prevent slipping, the top surface of each sensor was coated with 320-grit sandpaper. The sensors within each frame were spaced 3 cm apart in the mediolateral direction, and the hands were separated by ≈ 35–40 cm to ensure comfortable finger placement. Adjustments were made in the anterior–posterior direction to align precisely with the individual participants’ finger anatomy, ensuring consistency throughout the testing session. Participants maintained a steady hand position, ensuring fingertip contact with each sensor, and slightly flexed interphalangeal and metacarpophalangeal joints, resulting in a dome-shaped palm. The experimental setup is shown schematically in Fig. 2A.

Data acquisition and visual feedback were facilitated through a customized software based on the LabVIEW package (LabVIEW version 2014; National Instruments, Austin, TX, USA). Signals from the force sensors were collected, amplified, and sampled at 500 Hz using two 16-bit cards (PCI-6225, National Instruments, Austin, TX). Feedback was displayed on a 19″ monitor positioned 0.6 m in front of the participant at eye level (Fig. 2A).

### Procedures

Participants performed two main tasks, the maximum voluntary contraction (MVC) task and the main experimental trials, which consisted of Short trials (10-s long) and Long trials (60-s long). The MVC task was always performed first and used to set the force targets for the subsequent experimental trials.

#### Maximum voluntary contraction (MVC) task

Participants performed three MVC trials. In each trial, participants received visual feedback on the total pressing force (F_TOT_) generated by both hands combined, displayed as a vertical bar. Upon hearing an auditory “go” signal, participants increased their force, reaching maximal F_TOT_ within approximately 3 s. A rest period of at least 30 s followed each trial. The instruction was: “After the ‘go’ signal, gradually press with the index and middle fingers of both hands to your maximal force, hold that level for at least 0.5 s, then relax.” The highest force achieved across the three MVC trials (F_MVC_) was used to establish target force levels for the subsequent experimental tasks. We opted against measuring MVC for each hand and each finger to avoid extending the experimental procedure and potentially causing fatigue as a result of multiple MVC tests.

#### Experimental trials: short trials

Short trials involved three feedback conditions addressed further as “Feedback-Variable” conditions:

- **F**^**HAND**^** condition**: Visual feedback for the force produced by individual hands was provided by two black cursors, each moving vertically according to the force magnitude.
- **F**^**TOT**^** condition**: Visual feedback for the total force produced by both hands combined was provided by a single black cursor moving vertically according to the combined force magnitude.
- **F**^**TOT**^** + F**^**HAND**^** condition**: Visual feedback for individual hands and the combined total force was provided simultaneously by three black cursors moving vertically according to individual hand forces and total combined force output.

There were two types of feedback, “continuous” (FB_CONT_, the cursor showing the current force magnitude was seen at all times) and “off-target” (FB_OFF-TARGET_, the cursor was visible only when it was outside the margins of the target). These are addressed further as “Feedback-Type” conditions. As illustrated in Fig. 2B, the three targets were displayed continuously on the screen as either a red ring or a black filled circle corresponding to continuous and off-target feedback, respectively. We did not use conditions with feedback on the forces produced by each individual digit to avoid increasing the duration of the study and dealing with the accumulation of fatigue and boredom.

The F_TOT_ target was centered on the screen, while the two F_HAND_ targets were positioned 5 cm to the left and right sides of the center. The vertical coordinate of each target corresponded to the required force magnitude: 10% MVC for the left and right F_HAND_ targets and 20% MVC for the F_TOT_ target. These magnitudes were selected to match the total force magnitude in the conditions with different feedback. The diameter of each target corresponded to ± 1% MVC. This corresponded to the diameter of ≈ 13 mm on the screen. The vertical (y-axis) range of the screen spanned from -10% MVC to 40% MVC, ensuring full visibility of the force traces and targets in the center throughout the task.

At trial onset, participants were instructed to press with all four instructed fingers and hold the black cursor(s) within their respective targets. All three cursors initially appeared at 0% MVC (near the bottom of the screen—“Baseline” shown in Fig. 2B) at the same horizontal positions as their targets. Depending on the feedback condition, vertical cursor movement was restricted as follows:

**F**^**TOT**^: Only the central cursor moved with force change; the two lateral cursors remained fixed in the initial location.

**F**^**HAND**^: Only the two side cursors moved with force change; the central cursor remained fixed in the initial location.

**F**^**TOT**^** + F**^**HAND**^: all three cursors moved with force change.

Each condition comprised a block of 24 trials, with 5-s inter-trial intervals. Each trial was 10-s long. The feedback conditions were block-randomized across the subjects: The trials within-a-condition were presented as a block, while the order of the conditions was fully randomized.

#### Experimental trials: long trials

During the 60 s long trials, only the target corresponding to F_TOT_ (center target at 20% MVC force level) was visible. Vertical cursor displacement reflected the F_TOT_ magnitude throughout the trial. Additionally, visual feedback on force-sharing between the two hands was provided during the initial 5 s through horizontal cursor movement (the right panel in Fig. 2B). A 50:50 sharing target, aligned with the center of the screen, represented equal contribution from the left and right finger pairs. The cursor moved to the left when the left hand produced more force than the right, and to the right when the right hand produced more force than the left. The extreme left and right edges of the screen corresponded to ± 20% MVC asymmetry in force sharing between the two hands. After the first 5 s, the horizontal cursor position was frozen in the ideal location corresponding to perfect task performance, although the vertical motion continued to reflect F_TOT_. The cursor remained visible throughout the trial. Participants were explicitly instructed to continue producing force in the same manner (“keep doing whatever you have been doing”) for the remaining 55 s of each Long trial.

Randomization was performed in the following manner: Participants first performed trials under one of the feedback visibility conditions (either off-target or continuous, randomized across participants). Within that condition, participants completed the three Short trial conditions (F^HAND^, F^TOT^, and F^TOT^ + F^HAND^) in a randomized order, followed by the Long trials. A single Long trial was performed per condition to avoid fatigue. Subsequently, participants completed the same sequence of Short and Long trials under the other feedback visibility condition.

In addition to the described breaks between trials and conditions, the subjects were given additional rest when requested. At the conclusion of the experimental session, none of the subjects reported fatigue.

### Data processing

Data processing was conducted using customized MATLAB scripts (R2022b; MathWorks, Inc., Natick, MA, USA). All force data underwent digital low-pass filtering at 20 Hz using a fourth-order Butterworth filter with zero lag.

For the Short trials, analysis was conducted over a 1-s steady-state interval, from the 8th to 9th second of each 10-s trial to ensure that subjects always reached a steady state and to avoid edge effects. For the Long trials, analysis was performed over discrete time windows of 100-ms duration, spaced by 1-s intervals, beginning at the 6th second and continuing through the end of each 60-s trial. These particular values were selected to avoid major effects of sample non-independence. The specific values of the time windows (100 ms) and 1-s intervals between the windows were selected based on pilot analyses that showed no significant differences in any main outcome variables when shorter and longer windows were explored and when the time intervals between the windows varied by a factor of two in either direction. Another, practical, reason was to obtain enough data points for performing the UCM-based analysis while avoiding excessively long trials that could lead to fatigue. Trials were screened for outliers based on a criterion of mean ± 2 SD of the total force (F_TOT_) produced; no trials were excluded according to this criterion.

Analysis of F_TOT_ stabilization by co-varying contributions of F_HAND_ by the right and left hands and analysis of F_HAND_ stabilization by co-varying contributions of F_I_ and F_M_ of that finger pair were conducted within the uncontrolled manifold (UCM) hypothesis framework (Scholz and Schöner 1999). The latter analysis was performed for the F_IM_ produced by the right and left hands separately. The UCM was defined as the null-space of the Jacobian matrix, J = [1 1], which mapped small changes in individual effector forces on the performance variable. In particular, the forces produced by the individual fingers of each hand were mapped on the hand force, F_HAND,R_ and F_HAND,L_ (for the right and left hand, respectively), while the forces produced by each hand were mapped on F_TOT_. Under this definition, both the UCM and its orthogonal (ORT) complement were one-dimensional for each hand or hand combination analyzed, as shown in the upper panels of Fig. 1.

The synergy index (ΔV) was computed as the normalized difference between variance components along the UCM (V_UCM_) and orthogonal to it (V_ORT_), quantified per dimension in their corresponding spaces: ∆V = (V_UCM_ – V_ORT_)/(V_UCM_ + V_ORT_). This normalization method constrains ΔV within the range {– 1; + 1}. Positive ΔV values signify a force-stabilizing synergy, whereas ΔV ≤ 0 indicates the absence of such synergy. Fisher’s z-transformation was subsequently applied prior to parametric statistical analyses.

In addition to these components, the total variance (V_TOT_) was calculated as the sum of V_UCM_ and V_ORT_: V_TOT_ = V_UCM_ + V_ORT_.

This analysis was performed for each block of 24 Short trials for each condition and each subject separately (inter-trial analysis). The same analysis was also conducted over the 54 samples collected within each of the Long trials (inter-sample analysis within a trial). The main outcome variables were V_UCM_, V_ORT_, V_TOT_, and ∆V_Z_ (where ‘z’ stands for z-transformation).

### Statistics and hypothesis testing

All data are reported as means with standard errors or medians with quartiles, depending on distribution characteristics. Before conducting parametric analyses, data were tested for normality and sphericity. The synergy index (∆V_Z_) was z-transformed, while the variance components (V_UCM_, V_ORT_, and V_TOT_) were log-transformed to meet normality assumptions.

Repeated-measures ANOVA was the primary statistical method used to test the specific hypotheses. These included: the hierarchical trade-off between synergies (Hypothesis-1), the absence of F_TOT_-stabilizing synergy without F_TOT_ feedback (Hypothesis-2), the effect of continuous vs. off-target visual feedback (Hypothesis-3), and the comparison between inter-trial and within-a-trial variance structures (Hypothesis-4).

For inter-trial analyses, within-hand and between-hand comparisons were performed using three-way and two-way repeated-measures ANOVAs with the following factors:

Feedback-Variable (three levels: F^TOT^, F^HAND^, F^TOT^ + F^HAND^);

Feedback-Type (two levels: FB_CONT_, FB_OFF-TARGET_); and

Effector (two levels: right, left), only for within-hand analysis.

Separate ANOVAs were conducted on each of the variance components (V_UCM_, V_ORT_, V_TOT_) and the synergy index (∆V_Z_). To examine Hypothesis-1 regarding hierarchical trade-offs between synergy indices, effects of Feedback-Variable on the synergy index (∆V_Z_) and variance components (V_UCM_ and V_ORT_) were explored. In addition, regression analysis was performed between V_UCM_ at the F_TOT_ level and V_ORT_ at the F_HAND_ level. Hypothesis-2 was tested by contrasting effects of F^HAND^ and F^TOT^ feedback conditions on the synergy index and variance components computed for F_TOT_. Hypothesis-3 was tested by exploring the effects of Feedback-Type on the synergy index and variance components across conditions with different levels of Feedback-Variable. Hypothesis-4 was tested using the comparison between the synergy index values computed over blocks of short trials and over time windows within single long trials.

The intra-trial variance structure was examined using the same dependent variables (∆V_Z_, V_UCM_, and V_ORT_), with an additional factor, Analysis (two levels: inter-trial, intra-trial). The factor Feedback-Variable was excluded, as visual feedback was provided only on F_TOT_ during the intra-trial analysis. For analyzing ∆V_Z_, repeated-measures ANOVAs were conducted with the factors Analysis, Feedback-Type (FB_CONT_, FB_OFF-TARGET_), and Effector (right, left). To further compare the variance components across subspaces (UCM vs. ORT), a separate three-way ANOVA was performed with the factors Analysis, Feedback-Type, and Space (V_UCM_, V_ORT_).

Post-hoc pairwise comparisons (*t*-tests) were corrected using Bonferroni adjustments. The significance threshold was set at *P* < 0.05. Where *p* values were close to the threshold, effect sizes were reported as generalized eta squared (η^2^). All statistical analyses were conducted in R version 4.2.2 (2022-10-31) using the RStudio IDE (version 2023.06.0 + 421).

## Results

### Inter-trial analysis: force-stabilizing index, ∆V_Z_

**Fig. 3:**
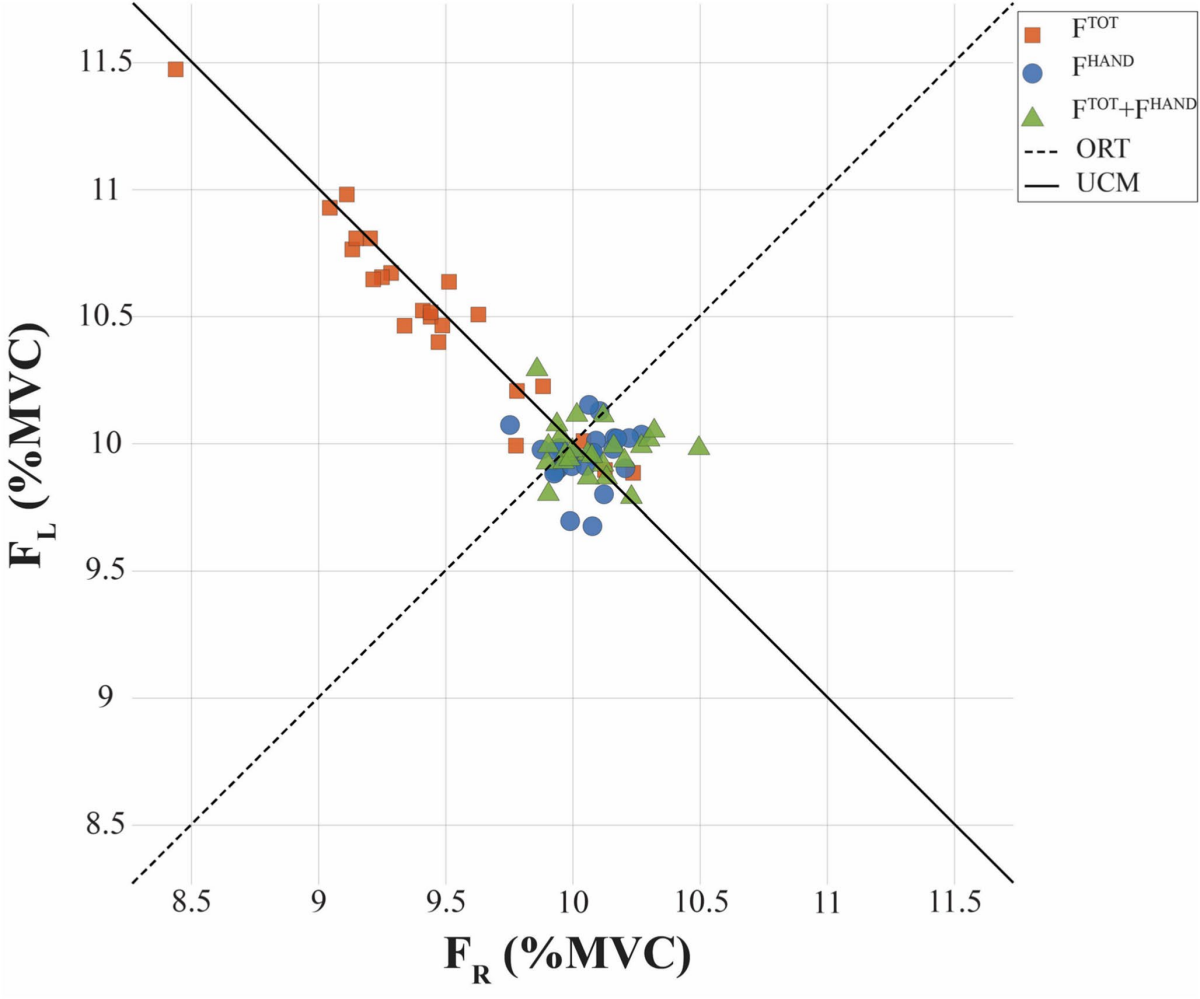
Short-trial data distribution for a representative subject under continuous visual feedback (FB_CONT_). Inter-trial scatters of the data points are shown on the left-hand force (F_L_, *y*-axis) versus right-hand force (F_R_, *x*-axis) plot across the target-feedback variables F^TOT^, F^HAND^, and F^TOT^ + F^HAND^

**Fig. 4:**
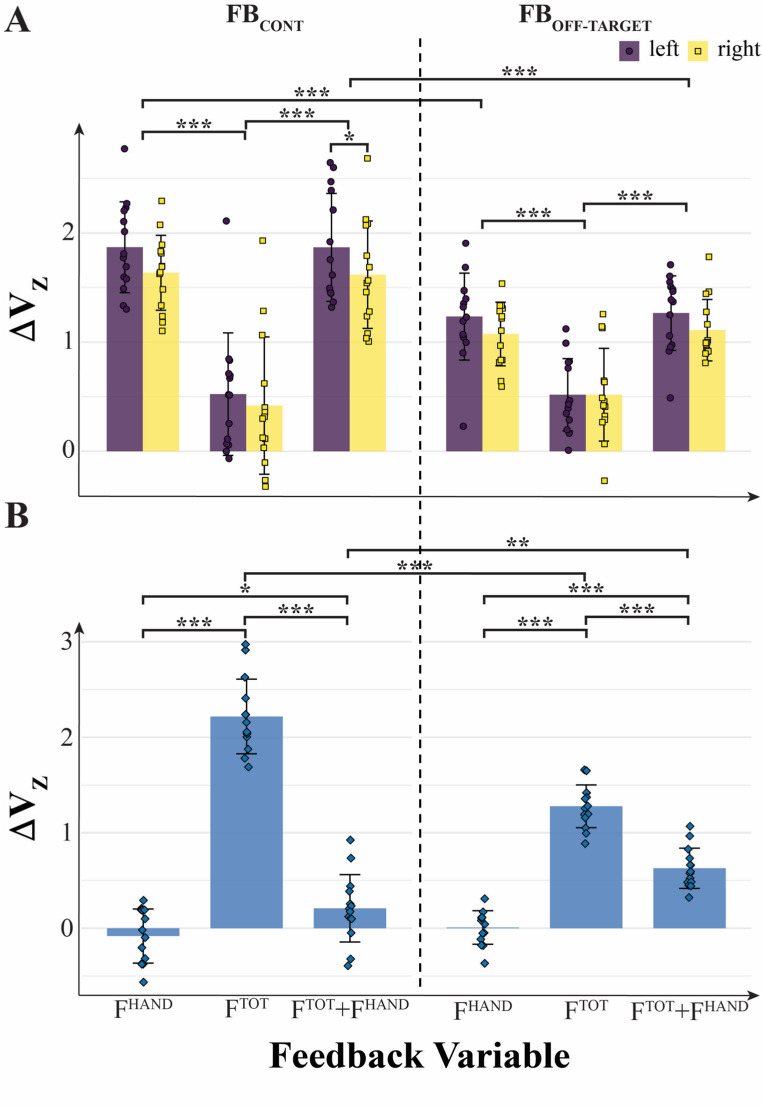
Normalized synergy index (ΔV_Z_) computed for short trials under different feedback conditions. **A** ΔV_Z_ values from within-hand analysis for the left and right hands. Note that synergy indices were higher when feedback was provided for individual hands (F^HAND^) or both individual and total force (F^TOT^ + F^HAND^), compared to other conditions. **B** ΔV_Z_ values from between-hand analysis combining both hands. Higher synergy indices were observed when feedback on total force (F^TOT^) was available. On the original scale, ΔV (median [Q1–Q3]) was: overall 0.763 [0.323–0.904]; by feedback-variable, F^TOT^: 0.627 [0.299–0.877], F^HAND^: 0.830 [0.142–0.920], F^TOT^ + F^HAND^: 0.781 [0.555–0.905]; by feedback type, FB_CONT_: 0.865 [0.206–0.956] and FB_OFF-TARGET_: 0.727 [0.416–0.850]. Here and in the captions for Figs. 5, 6 and 7, the values are pooled across the within-hand and between-hands analyses.

To test Hypotheses-1 and -2, we quantified the structure of inter-trial variance across the visual feedback conditions. Hypothesis-1 predicted a strong positive correlation across participants between V_UCM_ computed at the higher (F_TOT_) level and V_ORT_ computed at the lower (F_HAND_) level. Hypothesis-2 predicted force-stabilizing synergies only for force variables that received visual feedback. An example of data distributions across trials under conditions with continuous visual feedback on F^TOT^, F^HAND^, and both (F^TOT^ + F^HAND^) is presented in Fig. 3 for a representative subject. Sample trials in a similar task can be found in earlier publications (e.g., Fig. 2 in Solnik et al. 2015). Note the broad distribution of the data along the UCM (the solid slanted line) under F^TOT^ feedback, a smaller spread of the data along the UCM under the combined feedback condition, F^TOT^ + F^HAND^, and a nearly spherical distribution under the F^HAND^ feedback with about equal variability along the UCM and ORT directions. Such patterns were consistent across subjects. In particular, when visual feedback on F^TOT^ only was presented, the variance component (V_UCM_) that did not affect F_TOT_ was consistently higher than the variance (V_ORT_) affecting F_TOT_, resulting in positive values of ∆V_Z_. In contrast, under F^TOT^ visual feedback, the ∆V_Z_ for the contributing F_HAND_ was much smaller, V_UCM_ ≈ V_ORT,_ and ∆V_Z_ ≥ 0. Under F^HAND^ visual feedback, the situation reversed, V_UCM_ > V_ORT_, and ∆V_Z_ > 0 for F_HAND_, whereas the two variance indices for F_TOT_ were close in magnitude, and ∆V_Z_ was not significantly different from zero (∆V_Z_ ≈ 0). These effects supporting Hypotheses-1 and -2 can be seen clearly in Fig. 4, which presents the mean ∆V_Z_ data as well as the individual values.

The data for the combined feedback condition, F^TOT^ + F^HAND^, showed a more complex pattern related to Hypothesis-3 that stronger force-stabilizing synergies would be observed under continuous feedback as compared to the off-target feedback. When visual feedback was available only when the cursor was outside the target (the off-target condition), all three variables, F_TOT_ and both F_HAND_, showed ∆V_Z_ > 0 (V_UCM_ > V_ORT_). In contrast, when visual feedback was available continuously, ∆V_Z_ > 0 for F_HAND_ only, and V_UCM_ ≈ V_ORT_ (∆V_Z_ ≈ 0; not significantly different from 0) for F_TOT_.

These effects were confirmed by a three-way ANOVA on ∆V_Z_ (within-hand analysis: Feedback-Variable × Feedback-Type × Effector), which showed a significant main effect of Feedback-Type (F_[1, 13]_ = 29.88; *p* < 0.001, η^2^ = 0.165), confirming that ∆V_Z_ (continuous feedback) > ∆V_Z_ (off-target feedback) in support of Hypothesis-3. There were also significant main effects of Feedback-Variable (F_[2, 26]_ = 67.26; *p* < 0.001, η^2^ = 0.546) and Effector (F_[1, 13]_ = 5.60; *p* = 0.034; η^2^ = 0.032), and a significant two-way interaction Feedback-Variable × Feedback-Type (F_[2, 26]_ = 10.75; *p* < 0.001, η^2^ = 0.112), as shown in Fig. 4A.

For the between-hand analysis (Fig. 4B), the effects were confirmed by a two-way ANOVA on ∆V_Z_ (Feedback-Variable × Feedback-Type), which showed a significant main effect of Feedback-Variable (F_[2, 26]_ = 264.53; *p* < 0.001, η^2^ = 0.884) and a significant two-way interaction Feedback-Variable × Feedback-Type (F_[2, 26]_ = 61.87; *p* < 0.001, η^2^ = 0.529), but no main effect of Feedback-Type. Overall, Feedback-Type effects were less consistent compared to the effects of other main factors and provided only partial support for our Hypothesis-3.

Across all conditions, the force variables with visual feedback showed positive values of ∆V_Z_ in support of Hypothesis-2. The only exception was ∆V_Z_ not significantly different from zero for F_TOT_ under the F^TOT^ + F^HAND^ feedback presented continuously. Significantly smaller ∆V_Z_ values (t = 9.06, *p* < 0.001) were seen for F_HAND_ when F^TOT^ feedback was presented in the absence of F^HAND^ feedback. A symmetrical effect was seen for ∆V_Z_ computed for F_TOT_ (t = 14.17, *p* < 0.001): These values were the highest for the condition with F^TOT^ feedback.

### Inter-trial variance, V_ORT_

**Fig. 5:**
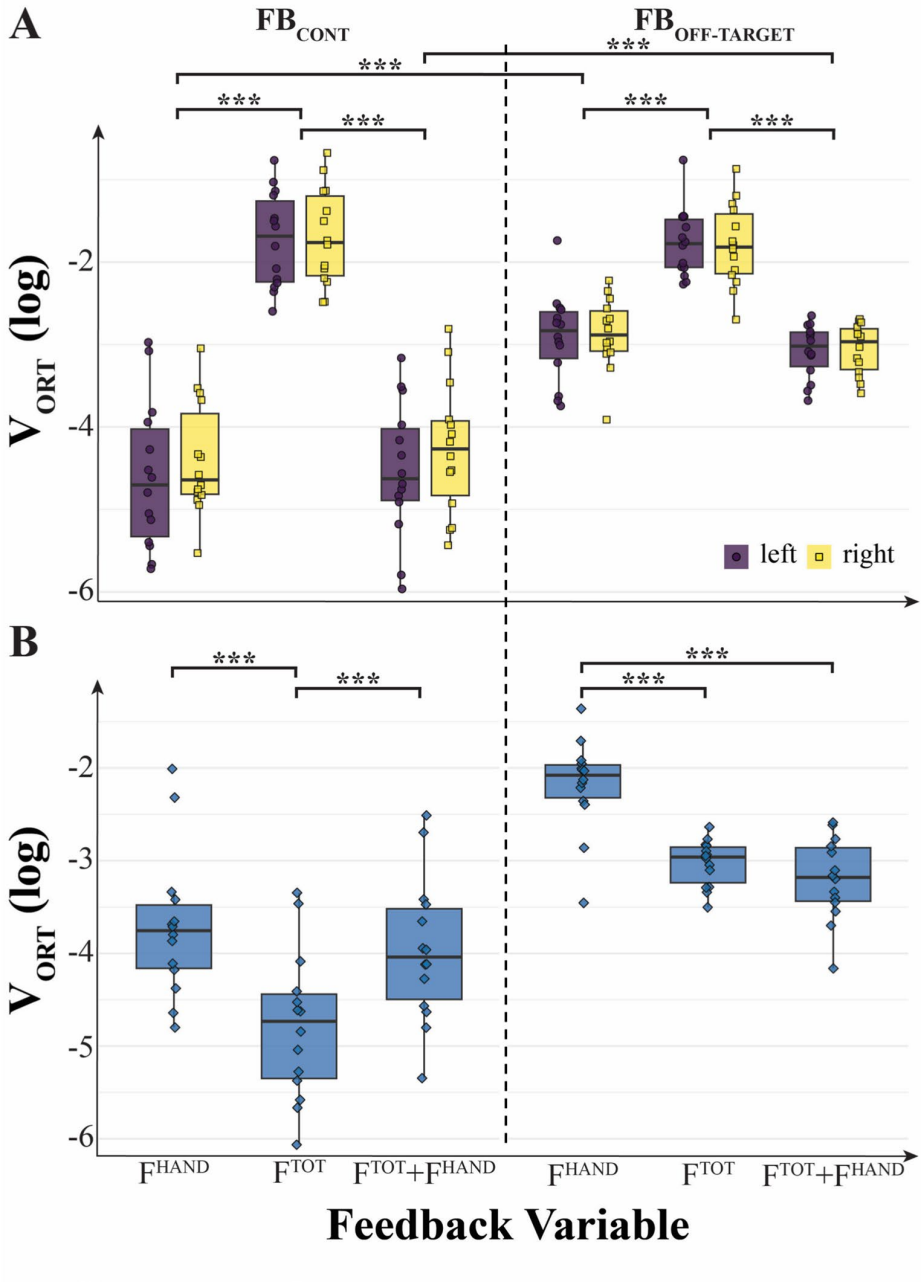
Log-transformed variance within the orthogonal subspace (V_ORT_) computed for short trials under different feedback conditions.**A** V_ORT_ values from within-hand analysis for the left and right hands. Variance was lower when feedback was provided for individual hands (F^HAND^) or for both individual and total force (F^TOT^ + F^HAND^), relative to other conditions. Additionally, V_ORT_ was reduced when feedback was presented continuously (FB_CONT_) compared to when it was given only upon deviation from the target (FB_OFF-TARGET_). **B** V_ORT_ values from between-hand analysis (combined hands). Lower V_ORT_ values were observed when total force feedback (F^TOT^) was available. Similar to the within-hand results, continuous feedback (FB_CONT_) resulted in reduced variance compared to feedback limited to outside-target deviations. On the original scale, V_ORT_ (median [Q1–Q3]) was: overall 0.043 [0.016–0.082]; F^TOT^: 0.075 [0.034–0.172], F^HAND^: 0.038 [0.016–0.092], F^TOT^ + F^HAND^: 0.031 [0.016–0.051]; by feedback type, FB_CONT_: 0.016 [0.009–0.039] and FB_OFF-TARGET_: 0.060 [0.044–0.112]

The described patterns in the synergy index magnitude, ∆V_Z_, could potentially reflect changes in either component of variance, V_UCM_ or V_ORT_, or in both. The effects of visual feedback on the index of variance affecting force magnitude (V_ORT_) showed a pattern, which looks like the inverse of the pattern for ∆V_Z_ (compare Fig. 4 with Fig. 5). Indeed, manipulations of feedback and effector with respect to which analysis was performed showed higher V_ORT_ values for conditions with smaller ∆V_Z_ values, and vice versa. In particular, V_ORT_ was consistently smaller for conditions with feedback on the corresponding force variable compared to other feedback conditions. As seen in Fig. 5, in between-hand analysis, V_ORT_ was smaller for F_TOT_, and in within-hand analysis, V_ORT_ was smaller for F_HAND_. These indices were also higher under the off-target feedback as compared to the continuous feedback condition.

For within-hand analysis (Fig. 5A), a three-way ANOVA on V_ORT_ (Feedback-Variable × Feedback-Type × Effector) showed a significant main effect of Feedback-Variable (F_[2, 26]_ = 241.57; *p* < 0.001, η^2^ = 0.718) and Feedback-Type (F_[1, 13]_ = 53.65; *p* < 0.001, η^2^ = 0.405), confirming that V_ORT_ (continuous feedback) < V_ORT_ (off-target feedback) and significant two-way interactions Feedback-Variable × Feedback-Type (F_[2, 26]_ = 41.33; *p* < 0.001, η^2^ = 0.285) and Feedback-Type × Effector (F_[1, 13]_ = 4.71; *p* = 0.049; η^2^ = 0.004). The effect of Feedback-Type is consistent with our Hypothesis-3.

For between-hand analysis (Fig. 5B), a two-way ANOVA on V_ORT_ (Feedback-Variable × Feedback-Type) showed significant main effects of Feedback-Type (F_[1, 13]_ = 55.41; *p* < 0.001, η^2^ = 0.554) and Feedback-Variable (F_[2, 26]_ = 44.14; *p* < 0.001, η^2^ = 0.302) and a significant two-way interaction between Feedback-Variable × Feedback-Type (F_[2, 26]_ = 19.83; *p* < 0.001, η^2^ = 0.108).

### Inter-trial variance, V_UCM_

The index of inter-trial variance with no effects on the corresponding force variables (V_UCM_) also showed modulation with the type of visual feedback and effector for which the index was computed. But this pattern was not identical to that for ∆V_Z_. In particular, consistent with Hypotheses-1 and -2, V_UCM_ for F_TOT_ was higher in the condition when feedback on F_TOT_ only was provided compared to the conditions when the F^HAND^ feedback was provided with or without F^TOT^ feedback. In contrast, V_UCM_ for each of the F_HAND_ variables showed little modulation with the type of visual feedback.

A three-way ANOVA on V_UCM_ was run for within-hand analysis (Feedback-Variable × Feedback-Type × Effector), which showed only a significant main effect of Feedback-Type (F_[1, 13]_ = 5.21; *p* = 0.04; η^2^ = 0.021). For between-hand analysis, a two-way ANOVA was run on V_UCM,_ Feedback-Variable × Feedback-Type. There were significant main effects of Feedback-Type (F_[1, 13]_ = 59.40; *p* < 0.001, η^2^ = 0.444) and Feedback-Variable (F_[2, 26]_ = 241.52; *p* < 0.001, η^2^ = 0.796) and significant two-way interaction Feedback-Variable × Feedback-Type (F_[2, 26]_ = 43.78; *p* < 0.001, η^2^ = 0.334). The effect of Feedback-Type on V_UCM_ may be viewed as unexpected (cf. Hypothesis-3) and requiring further exploration.

**Fig. 6:**
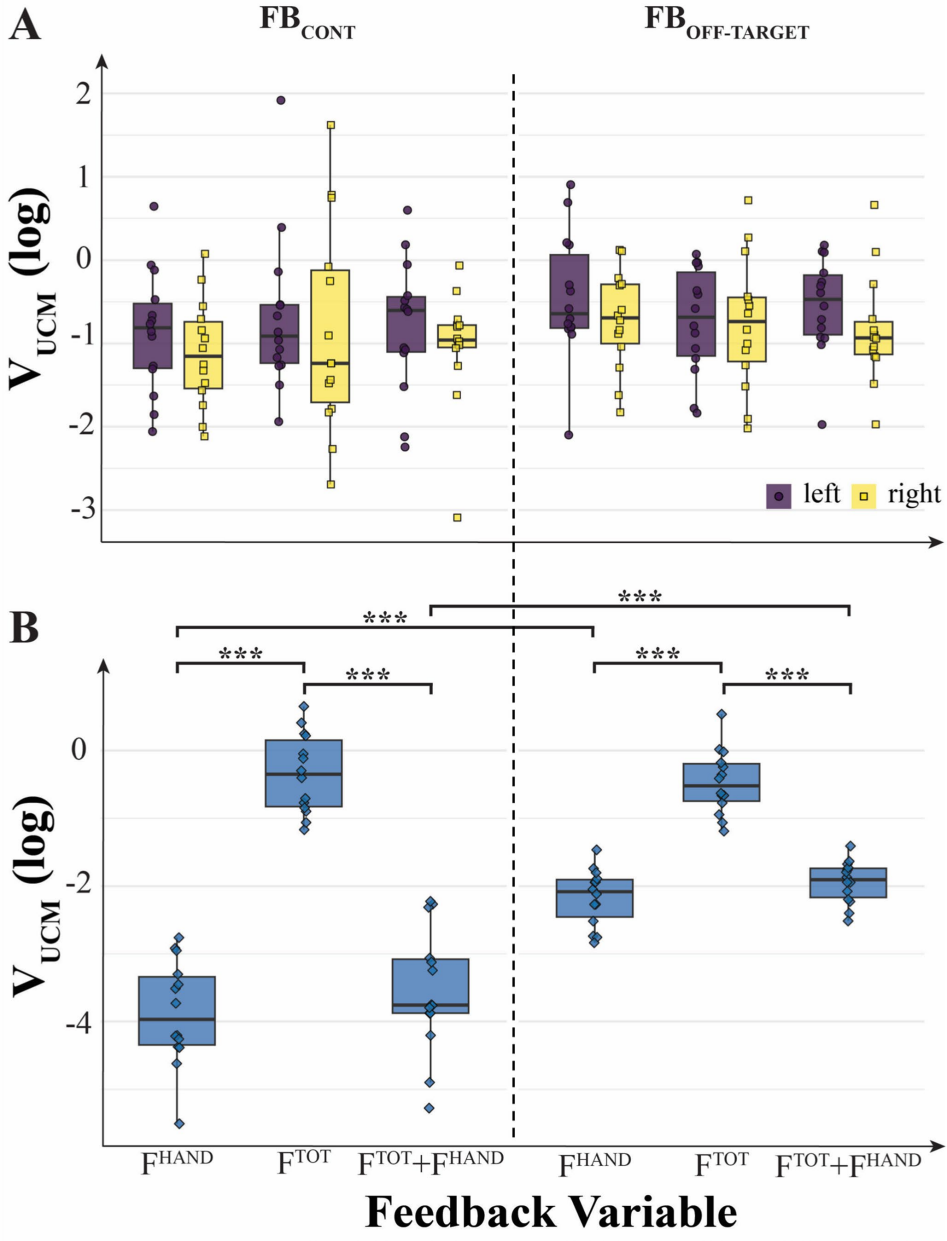
Log-transformed variance within the uncontrolled manifold subspace (V_UCM_) computed for short trials under different feedback conditions. **A** V_UCM_ values from within-hand analysis for the left and right hands. Variance was lower when feedback was provided continuously (FB_CONT_) compared to when it was delivered only upon deviation from the target (FB_OFF-TARGET_). However, no main effects of feedback variables were observed. **B** V_UCM_ values from between-hand analysis (combined hands). Lower variance was found when feedback was provided for individual hand forces. Differences between feedback types (FB_CONT_ vs FB_OFF-TARGET_) were evident only under conditions where feedback for each hand was available. On the original scale, V_UCM_ (median [Q1–Q3]) was: overall: 0.345 [0.140–0.668]; F^TOT^: 0.563 [0.344–0.892], F^HAND^: 0.187 [0.062–0.497], F^TOT^ + F^HAND^: 0.194 [0.097–0.476]; by feedback type, FB_CONT_: 0.318 [0.046–0.535] and FB_OFF-TARGET_: 0.401 [0.166–0.687]

These results are illustrated in Fig. 6, which shows no pairwise difference in V_UCM_ for the F_HAND_ for each of the hands under the F^TOT^ feedback and F^HAND^ feedback. In contrast, V_UCM_ for F_TOT_ was the largest under the ^FTOT^ feedback condition compared to both F^HAND^ and (F^TOT^ + F^HAND^) conditions (*p* < 0.001).

### Inter-trial total variance, V_TOT_

**Fig. 7:**
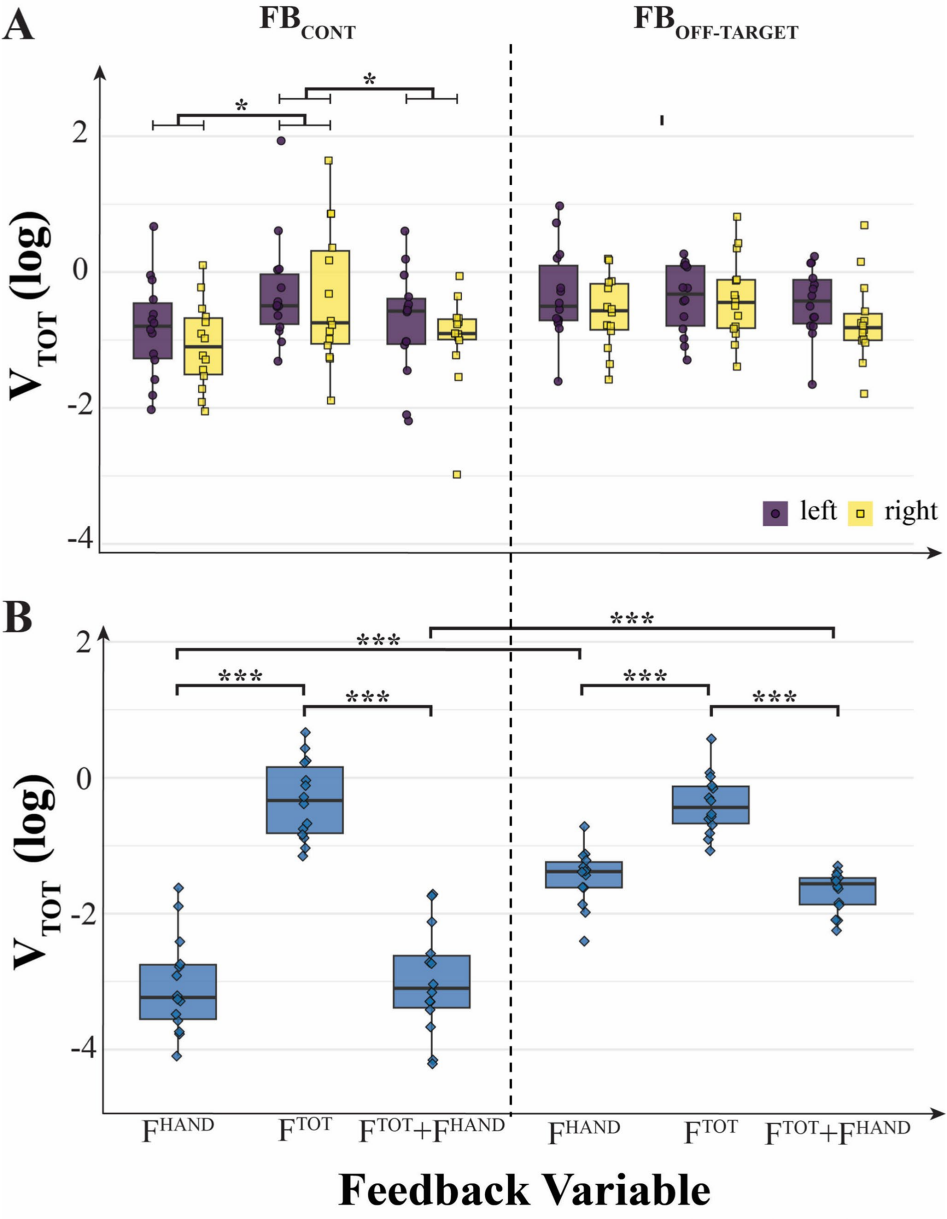
Log-transformed total variance within the uncontrolled manifold (V_UCM_) and along the orthogonal subspace (V_ORT_) computed for short trials under different feedback conditions. **A** Total variance (V_TOT_) from within-hand analysis for the left and right hands. **B** V_TOT_ values from between-hand analysis (combined hands). Across both analyses, the pattern of results resembled that of V_UCM_, with variance along the UCM subspace (V_UCM_) substantially exceeding that along the orthogonal subspace (V_ORT_), i.e., V_UCM_ ≫ V_ORT_. On the original scale, V_TOT_ (median [Q1–Q3]) was: overall: 0.411 [0.202–0.735]; F^TOT^: 0.697 [0.440–1.031], F^HAND^: 0.267 [0.147–0.542], F^TOT^ + F^HAND^: 0.245 [0.122–0.500]; by feedback type, FB_CONT_: 0.368 [0.073–0.658] and FB_OFF-TARGET_: 0.468 [0.257–0.783]

The mirror-like patterns of changes in V_UCM_ and V_ORT_ across the feedback conditions (see Figs. 5 and 6) suggest that the total variance, V_TOT_, could remain unchanged under at least some of the feedback manipulations. Figure 7 illustrates the average values of V_TOT_ and individual data across the feedback conditions. Note the visible modulation of V_TOT_ computed at the F_TOT_ level across conditions with different variables presented as feedback and the differences between the continuous and off-target feedback conditions (Fig. 7B). Namely, when the F^TOT^ visual target was presented, V_TOT_ was the largest under both continuous and off-target feedback conditions. Presenting F^HAND^ targets, with or without the F^TOT^ target, led to a major drop in V_TOT_, which was larger for the continuous feedback condition.

These patterns were confirmed by a two-way ANOVA on V_TOT_, Feedback-Variable × Feedback-Type, which showed both significant main effects (Feedback-Variable: F_[2,26]_ = 240.36; *p* < 0.001, η^2^ = 0.738; Feedback-Type: F_[1,13]_ = 52.70; *p* < 0.001, η^2^ = 0.430) and a significant two-way interaction (F_[2,26]_ = 41.72; *p* < 0.001, η^2^ = 0.305). Pairwise contrasts confirmed significant differences between the F^TOT^ and F^HAND^ feedback conditions and between F^TOT^ and F^TOT^ + F^HAND^ conditions (p < 0.001) without a difference between the F^HAND^ and F^TOT^ + F^HAND^ conditions (*p* = 1).

In contrast, analysis of V_TOT_ at the lower (F_HAND_) level showed similar but minor effects of changes in the target and type of visual feedback (Fig. 7A). There were also no visible differences between the Effectors (right and left hands). ANOVA on V_TOT_ (Feedback-Variable × Feedback-Type × Effector) showed significant main effects of Feedback-Variable (F_[2,26]_ = 3.51; *p* = 0.045; η^2^ = 0.059) and Feedback-Type (F_[1,13]_ = 6.53; *p* = 0.024; η^2^ = 0.031) and no interactions. Pairwise contrasts confirmed significant differences between the F^TOT^ and F^HAND^ feedback conditions (*p* = 0.044) and between F^TOT^ and F^TOT^ + F^HAND^ conditions (*p* = 0.01), as well as significant differences between the Feedback-Types: FB_CONT_ vs FB_OFF-TARGET_ (*p* = 0.007).

### Analysis of the trade-off between the levels

**Fig. 8:**
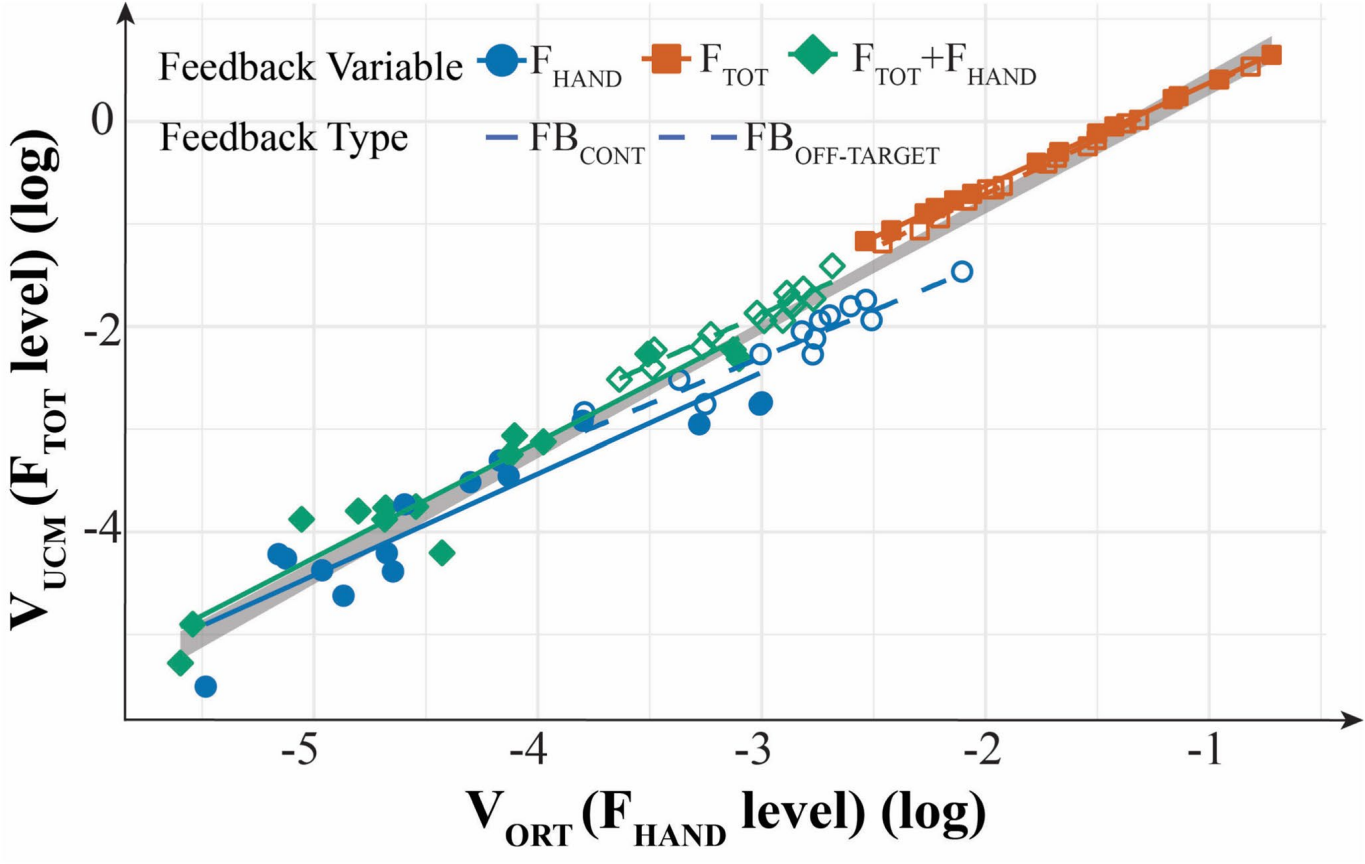
Correlation between the variance within the uncontrolled manifold (V_UCM_) at the upper task level (F_TOT_) and the variance along the orthogonal subspace (V_ORT_) at the lower level (F_HAND_), indicating a trade-off between synergy indices across control levels. Note the high coefficients of determination (R^2^) for each regression line, as well as for the overall regression across all feedback conditions

As suggested by Hypothesis 1 in the Introduction, the trade-off between the synergy indices (∆V_Z_, see Fig. 4) computed at the upper level (F_TOT_ level) and lower level (F_HAND_ level) originates from an inherent correlation between V_UCM_ at the F_TOT_ level and V_ORT_ at the F_HAND_ level. The results of this analysis are illustrated in Fig. 8, which shows the values of V_UCM_ at the F_TOT_ level as functions of V_ORT_ at the F_HAND_ level across individual subjects and feedback conditions. Note that, within each condition, there was a significant positive correlation between these two variables (R > 0.9; *p* < 0.01 for each regression). Moreover, the regression lines nearly overlap and cover the whole range of changes in the two variance variables. The different locations of the data clusters reflect the effects of feedback described in earlier subsections of the Results.

### Within-a-trial analysis

This analysis was run, in particular, to test Hypothesis-4, which predicted lower synergy index values for within-a-trial analysis as compared to the inter-trial analysis. During the within-a-trial analysis, ∆V_Z_ for F_TOT_ estimated across the samples was consistently positive for both feedback types—continuous and off-target. This index, however, was significantly smaller than in the inter-trial analysis, on average 0.81 ± 0.48 vsersu 1.75 ± 0.57, in support of Hypothesis-4. This finding has been supported by the two-way ANOVA, Analysis × Feedback-Type, which showed the main effect of Analysis (F_[1, 13]_ = 68.10; *p* < 0.001, η^2^ = 0.583), Feedback-Type (F_[1, 13]_ = 23.63; *p* < 0.001, η^2^ = 0.310), and two-way interaction (F_[1, 13]_ = 21.10; *p* < 0.001, η^2^ = 0.210).

For the within-hand analysis, a three-way ANOVA on ∆V_Z_ (Analysis × Feedback-Type × Effector), showed a significant main effect of Effector (F_[1, 13]_ = 6.57; *p* = 0.024; η^2^ = 0.034) and a significant Analysis × Effector interaction (F_[1, 13]_ = 4.75; *p* = 0.048; η^2^ = 0.02). Pairwise comparison confirmed that ∆V_Z_ for IM_L_ > IM_R_ (*p* = 0.007). Note that this effector difference was present only in the intra‐trial analysis (*p* = 0.006) and not in the inter‐trial analysis (*p* = 0.591).

**Fig. 9:**
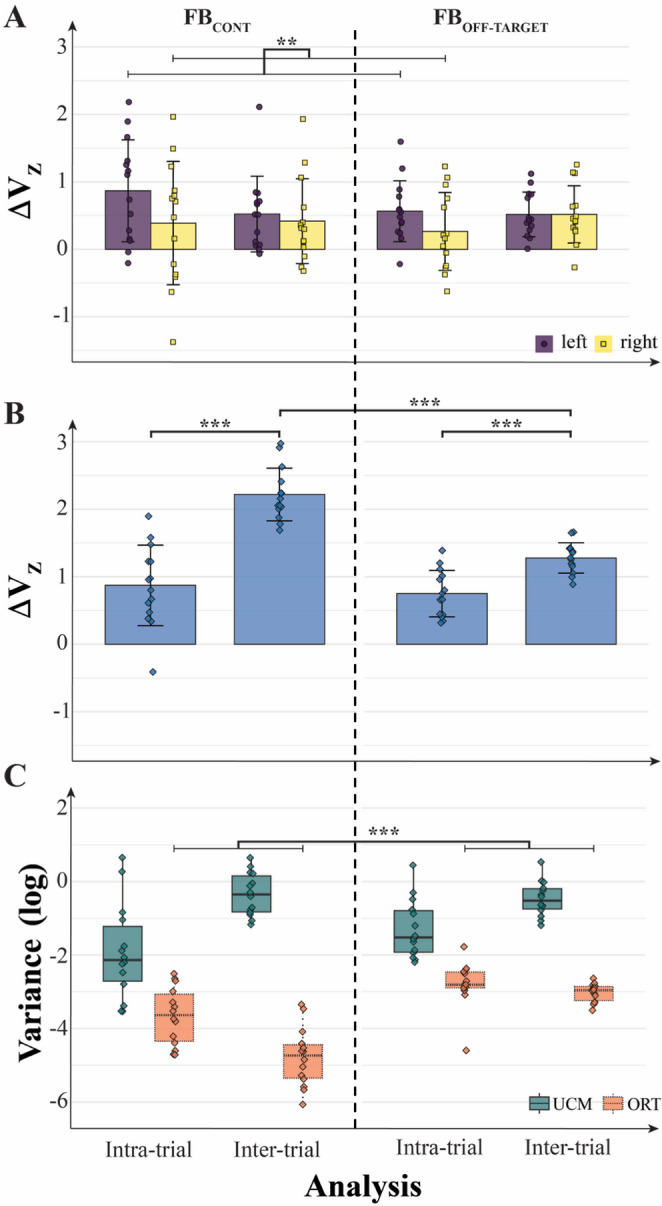
Synergy indices and variance components computed for different analysis types (inter-trial vs intra-trial) and feedback conditions. **A** Normalized synergy index (ΔV_Z_) computed separately for each hand. Differences between the left and right hands emerged only in the intra-trial analysis. **B** ΔV_Z_ values from between-hand analysis (combined hands). Differences between feedback types (FB_CONT_ vs FB_OFF-TARGET_) were observed only in the inter-trial analysis. **C** Log-transformed variance within the UCM (V_UCM_) and orthogonal (V_ORT_) subspaces. An increase in V_ORT_ was observed under the FB_OFF-TARGET_ condition, with no corresponding change in V_UCM_

**Fig. 10:**
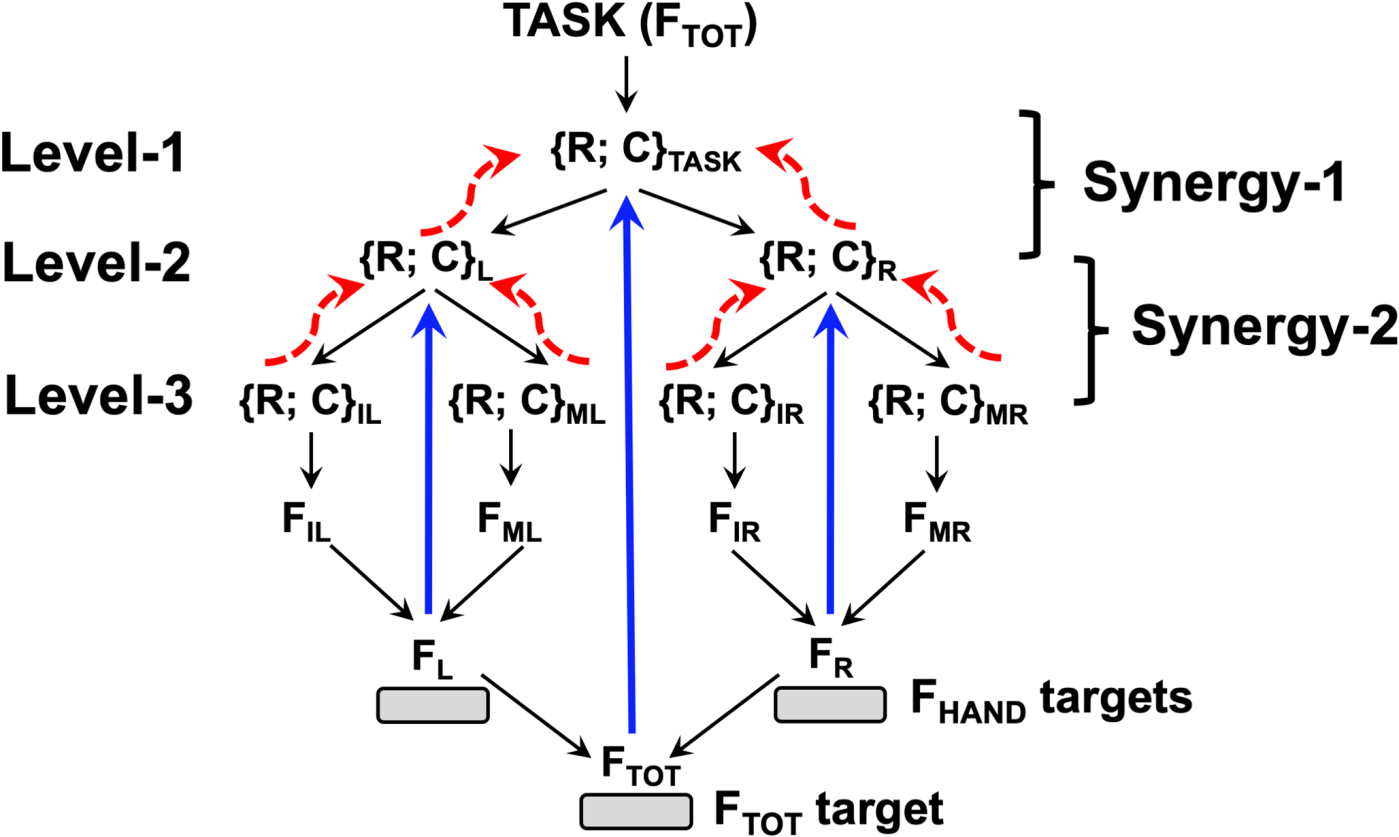
Overall schematics of the presumed control of the four-finger task involving two hands. The reciprocal and coactivation commands, {R; C} emerge at three levels, those of the task force (Level-1), hand force (Level-2) and finger force (Level-3). The back-coupling loops (dashed thick arrows, red) and visual feedback (thick straight arrows, blue) contribute to synergies ensuring stability of the performance by co-varying adjustments of commands. Note that specifying targets for individual hands only effectively removes all processes involving Level-1 and is expected to lead to no synergy stabilizing total force. Subscripts: TOT–total, R–right; L–left: I–index finger; M–middle finger

To analyze the variance components across the two spaces, UCM and ORT, we conducted a between-hand analysis using a three-way ANOVA (Analysis × Feedback-Type × Space). This showed significant main effects of Feedback-Type (F_[1, 13]_ = 87.13; *p* < 0.001, η^2^ = 0.223) and Space (F_[1, 13]_ = 419.83; *p* < 0.001, η^2^ = 0.755), as well as significant two-way interactions between Feedback-Type × Space (F_[1, 13]_ = 23.63; *p* < 0.001, η^2^ = 0.117) and Analysis × Space (F_[1, 13]_ = 68.10; *p* < 0.001, η^2^ = 0.293), and a three-way interaction (F_[1, 13]_ = 21.10; *p* < 0.001, η^2^ = 0.073). Post-hoc pairwise tests indicated that, in the off-target feedback condition, both intra-trial and inter-trial variance indices for F_TOT_ were larger than under the continuous feedback condition (*p* < 0.001) (Fig. 9) and that only V_ORT_, not V_UCM_, changed with Feedback-Type (*p* < 0.001) (Fig. 10).

## Discussion

All the formulated specific hypotheses have received support in the experiment, although some of them have been supported only partly. In particular, in support of Hypothesis-1, we observed strong positive correlations across participants between V_UCM_ computed at the higher (F_TOT_) level and V_ORT_ computed at the lower (F_HAND_) level across all the visual feedback conditions (Fig. 8 in Results). While the correlation does not necessarily imply causation, these effects are expected from Hypothesis-1. Note that these conditions were associated with broadly varying values of the synergy index. Additional support for Hypothesis-1 was received by observations of F_TOT_-stabilizing synergies across conditions with F^TOT^ feedback, while in conditions with F^HAND^ feedback, we observed F_HAND_-stabilizing synergies, as reflected in the inequality V_UCM_ > V_ORT_ and ∆V > 0. In contrast, no such synergies (∆V ≈ 0) were seen in conditions when a particular force variable, F_TOT_ or F_HAND_, received no visual feedback, even when its magnitude was indirectly constrained, as was the case for F_TOT_ in the condition with F^HAND^ feedback (Figs. 4, 5 and 6 in Results; cf. Hypothesis-2). Overall, these results can be seen as a reflection of a trade-off between synergies at different hierarchical levels illustrated in Fig. 1 in the Introduction (see also earlier studies by Gorniak et al. 2007, 2009).

Analysis of inter-sample variance indices within single trials with the sharing pattern close to the one preferred naturally by the subjects (50:50), provided support for Hypothesis-4. The synergy index under this condition was lower compared to the inter-trial analysis (Fig. 9 in Results). These results corroborate the hypothesis that V_UCM_ can be viewed as produced by two contributors, inter-trial sharing differences (V_UCM,SH_) *across trials*, which is obviously absent in the *within-a-trial* analysis, and within-a-trial covariation constraining the variability of the elements to the UCM that takes place over time during individual trials (V_UCM,CoV_), and both play an important role in defining the structure of variance (see also Abolins et al. 2025a, b).

Our Hypothesis-3 was related to the role of continuous vs. intermittent (i.e., off-target) visual feedback. In line with this hypothesis, we observed significantly higher V_ORT_ values in the “off-target” feedback condition, contributing to the smaller ∆V indices. These effects were significant only in the within-hand analyses, not as a main effect in the between-hand analyses. Interestingly, the data were tightly clustered across subjects in the off-target feedback condition compared to the continuous condition (Figs. 5, 6 and 7), suggesting that allowing errors in performance smaller than the target size can lead to higher consistency across subjects. The differences in V_UCM_ between the continuous and intermittent feedback conditions were relatively small, however, suggesting that the feedback type affected primarily only one of the variance components, namely, along the ORT direction (as shown in Fig. 5 in Results).

### Two sources of variance within the UCM

Although variance within the UCM, by definition, has no effect on the magnitude of the salient performance variable, it reflects the stability of that variable by showing how efficient the CNS is in channeling unavoidable variations in elemental variables into the UCM. The importance of V_UCM_ has been demonstrated in studies of neurological patients with impaired control of action stability. In particular, changes in the index of action stability during steady-state performance (∆V) between patients with multiple sclerosis and age-matched controls have been shown to reflect differences in V_UCM_, not V_ORT_ (Jo et al. 2016). In that study, the group differences in the magnitude of anticipatory synergy adjustments (ASAs, cf. Olafsdottir et al. 2005) in preparation for a quick action were also associated with differences in the changes in V_UCM_. Patients with Parkinson’s disease on- and off- dopamine-replacement drugs showed pronounced differences in the characteristics of V_UCM_, not V_ORT_ (Falaki et al. 2017). Along similar lines, training in conditions challenging action stability led to a significant increase in V_UCM_ in both young and older persons, whereas V_ORT_ dropped in both groups (Wu et al. 2012, 2013).

Earlier models related the structure of inter-trial variance to both feedback-based and feed-forward circuits (Latash et al. 2005; Goodman and Latash 2006). Both possibilities have been combined into a scheme on the production of multi-element actions (Martin et al. 2009, 2019). Recent studies have offered a method to quantify the contributions of two components to V_UCM_ (and ∆V), which have been tentatively associated with the feed-forward and feedback circuitry, by separating the effects of inter-trial variation of the sharing pattern and intra-trial co-variation of elemental variables (Solnik et al. 2015; Abolins et al. 2025a, b). We built on those studies to explore how this scheme can handle tasks where each of the effectors represents a combination of lower-level elements, and by expanding the range of feedback conditions, in particular by contrasting behaviors under continuous and intermittent (off-target) feedback.

Our findings can be interpreted within the scheme of control with two basic commands, reciprocal and coactivation (*R* and *C*), at different levels of a task-specific control hierarchy (reviewed in Latash 2010; Feldman 2015). This scheme is presented schematically in Fig. 9 for the task of producing forces with the index and middle fingers of each hand, as in our experiment. Note that there are two kinds of back-coupling loops in this scheme to the control levels: From peripheral sensory endings reflecting the performance (primarily visual feedback in our study, with possible contributions from force-sensitive endings in the hand) and from the lower (hand-specific, F_HAND_) control level to the higher (total force, F_TOT_) level. This scheme has several non-trivial predictions related to the findings in our study. First, if all the back-coupling loops function, their effects are expected to lead to the synergic inequality, V_UCM_ > V_ORT_, for the task-specific variable. If no target for F_TOT_ is presented, however, the task control level shifts to the lower level of the hierarchy, and {*R*; *C*} pairs are specified for each hand separately. Then, the back-coupling loops to the level related to specification of F_TOT_ stop functioning, and no synergy stabilizing F_TOT_ is expected (V_UCM_ ≈ V_ORT_) even though F_TOT_ variability can be low due to the target-related constraints on each F_HAND_. In contrast, if only the F_TOT_ target is presented, V_UCM_ ≈ V_ORT_ for each of the finger pairs is expected. The relatively high V_ORT_ leads to sufficient variability in the contributions of each of the finger pairs to F_TOT,_ thus facilitating the emergence of F_TOT_-stabilizing synergies (cf. Gorniak et al. 2007).

When all three targets are presented, however, all three of the performance variables can be stabilized by the involved back-coupling loops, two F_HAND_ variables, and F_TOT_. Given the inherent competition between synergies at different hierarchical levels (see the next section), the synergy indices for all three performance variables are expected to be lower compared to their values when visual targets for only F_HAND_ or only F_TOT_ are presented (see Fig. 4 in Results). Subtle manipulations of the feedback, however, can sway this competition in favor of F_TOT_ or in favor of F_HAND_, as we observed in the comparison of the conditions with continuous and off-target feedback. In particular, under continuous visual feedback, the subjects were accurate in keeping the cursor close to the centers of the targets, which led to relatively small variance at the F_HAND_ level, leaving little room for having large enough V_UCM_ at the F_TOT_ level. Indeed, much smaller V_UCM_ values were seen under the continuous feedback compared to the off-target feedback condition, confirming this interpretation.

We observed differences in the synergy index between the left and right hands, which were under the significance level but were typically in the direction of higher ∆V values in the left (non-dominant) hand (Fig. 4 in Results). These differences are consistent with earlier observations reporting stronger force-stabilizing synergies during steady force production by the non-dominant hand (Park et al. 2012; de Freitas et al. 2019). They are also in line with the dynamical dominance hypothesis (Sainburg 2005) suggesting better performance of the non-dominant hand in steady-state tasks, such as holding a loaf of bread, and better performance of the dominant hand in movement tasks, such as moving the knife to slice a piece of bread.

### Hierarchical control with referent coordinates

The concept of hierarchical control of movements can be dated back at least to the classical studies of Hughlings Jackson (1889), who introduced the concept of multiple cortical representations of motor elements, from single muscles to the whole body. This concept is also central for the theory on the construction of movements by Bernstein (1947), who considered all actions as being built on at least two levels, the leading level and the background level(s). Within the aforementioned theory of control with spatial referent coordinates for effectors, the control at the task level is assumed to be relatively low-dimensional, representing two basic command vectors, reciprocal and coactivation (*R* and *C*), which are defined as functions of spatial referent coordinates for the agonist and antagonist muscle groups. The implementation of any action involves a sequence of few-to-many mappings leading to the emergence of higher-dimensional sets of the *R*- and *C*-commands at the levels of the involved effectors, such as limbs and joints, and, ultimately, to time profiles of high-dimensional sets of thresholds of the stretch reflex (λ, cf. Feldman 1986) for the involved muscles and motor units.

Potentially, the output at any effector level can be stabilized by co-varied adjustments of the respective {*R*; *C*} pairs (Ambike et al. 2016; Nardon et al. 2022). The action of individual muscles can be stabilized by co-varied involvement of motor unit groups (MU-modes), likely based on spinal circuitry (Madarshahian et al. 2021; Latash et al. 2023). During actions performed by large effectors up to the whole body, task-specific performance variables can be stabilized by co-varied involvement of stable muscle groups addressed as factors, primitive, or modes (reviewed in Ivanenko et al. 2006; Giszter 2015; Overduin et al. 2015; Latash 2020). Such hierarchical control has also been considered at the levels of kinetic or kinematic variables produced by individual elements involved in a multi-element task. For example, during prehensile tasks, a hierarchy has been suggested (Arbib et al. 1985) with the task-related force and moment vectors applied to the hand-held object shared between the thumb and an opposing “virtual finger”, an imaginary digit with the mechanical action equivalent to that of the actual fingers combined. At the lower level of the hierarchy, the action of the virtual finger is shared among the actual fingers. During two-arm pointing tasks, the control has been considered at the level of joint rotations moving the endpoint of each of the arms as well as at the level of the vectorial or scalar distance between the endpoints (Domkin et al. 2002, 2005).

There is, however, a catch inherent to hierarchical control schemes: A high ∆V index requires relatively high V_UCM_ magnitudes to satisfy V_UCM_ > V_ORT_, which requires relatively high variability of the contributing elemental variables. At the level of analysis of each of the elemental variables, this is expected to translate into high values of V_ORT_, which makes it hard or impossible to satisfy the inequality V_UCM_ > V_ORT_, i.e., to have a high synergy index. Along similar lines, a performance variable stabilized at a lower level can serve as an elemental variable at a hierarchically higher level. If this elemental performance variable is highly stable, its variations are constrained, which does not allow it to show large enough variability that may be needed to satisfy the inequality V_UCM_ > V_ORT_ at the higher level of analysis.

In our experiment, we observed several examples of the trade-off between force-stabilizing synergies at the F_HAND_ and F_TOT_ levels. The general pattern was similar for the synergy index, ∆V, computed for F_HAND_ and F_TOT_: Large positive values for variables with visual feedback and low (close to zero) values for variables without visual feedback (Fig. 4 in Results). However, at the upper level of the hierarchy, the modulation of ∆V for F_TOT_ with visual feedback was primarily due to the modulation of V_UCM_, whereas at the lower level of the hierarchy, the modulation of ∆V for F_HAND_ was primarily due to the modulation of V_ORT_ (see Figs. 5 and 6). This pattern was particularly obvious under continuous visual feedback on the force variables.

Additional insights were provided by the analysis of total variance (V_TOT_). At the F_HAND_ level, V_TOT_ showed no significant effects of the factors reflecting visual feedback and was dominated by V_UCM_ across all conditions, which also showed no significant effects of feedback manipulations (see Fig. 7). In contrast, the V_ORT_ magnitude was significantly smaller when F^HAND^ feedback was available and smaller for the continuous feedback compared to the off-target feedback (see Fig. 5). As a result, changes in V_ORT_ were the main factor affecting ∆V across the feedback conditions. At the F_TOT_ level, V_TOT_ depended strongly on feedback manipulations, which affected both V_UCM_ and V_ORT_ (Figs. 5, 6 and 7). In particular, both V_TOT_ and V_UCM_ were much smaller when F^HAND^ feedback was available (even in combination with F^TOT^ feedback!). This effect was particularly strong under the continuous feedback condition. The comparison of the effects suggests that, in agreement with the described trade-off, changes in V_ORT_ at the F_HAND_ level with feedback manipulation were the dominant factor affecting, in particular, changes in V_UCM_ at the hierarchically higher F_TOT_ level (cf. Hypothesis-1 in the Introduction).

A number of earlier studies provided experimental support for the trade-off between the synergy index magnitudes computed at two levels of a hierarchy (Gorniak et al. 2007, 2009). The described interpretation, however, remained speculative. Our study is the first to demonstrate significant correlations between V_UCM_ computed at the higher (F_TOT_) level and V_ORT_ computed at the lower (F_HAND_) level of the hierarchy across the participants (Fig. 8 in Results). In other words, subjects who facilitated relatively sloppy performance at the lower level were more likely to have higher V_UCM_ and stronger synergies at the higher level. A number of studies have provided evidence that can be seen as specific illustrations of this general rule, i.e., facilitating higher variance at the level of elements to be able to assemble task-specific synergies. These involved studies of kinematic synergies during throwing the basketball into the basket (Hasanbarani and Latash 2020) and during walking with and without stepping targets (Rosenblatt et al. 2014), as well as studies of the effects of practice challenging performance stability (Wu et al. 2012, 2013).

We have to admit that manipulating the number of salient visual targets and of the variables reflected in the visual feedback could exert effects at higher levels of the control. These factors could lead to sharing attention between the targets. In addition, the subjects could change their understanding of the task goals between focusing on individual hand force production to combined F_TOT_ production. These factors could, by themselves, have effects on the stability of performance as reflected in our main outcome variable, such as V_UCM_, V_ORT_, and ∆V. At this moment, we cannot disambiguate such effects from the ones discussed in this section and related to the neural control within a hierarchical system. While there is qualitative similarity of the effects observed in our study and in earlier studies by Gorniak et al. (2007, 2009), which did not manipulate the number of targets, additional study may be needed to address the issues of sharing attention across effectors and targets and of switching task goals when visual feedback conditions are modified. These issues relate to the levels C and D within the hierarchical control scheme introduced by Bernstein (1947) and pertain more to the field of ecological psychology (cf. Gibson 1979; Turvey 2007).

### Continuous versus intermittent control of force

Force production tasks are particularly sensitive to the availability and quality of visual feedback. In particular, when visual feedback becomes unavailable, the force magnitude drifts, typically to lower values, and the performer is unaware of the drift even when it reaches substantial magnitudes, up to 30–40% of the initial force level (Vaillancourt and Russell 2002; Ambike et al. 2015). These phenomena have been interpreted as reflecting the loss of stability of the {*R*; *C*} control variables at the task level (Abolins and Latash 2022). Drifts in the *C*-command have been documented and interpreted as primary factors causing the force drifts (Reschechtko and Latash 2017; Cuadra et al. 2021). Force drifts can be seen as natural consequences of partial loss of stability of the force magnitude. This interpretation is corroborated by earlier studies exploring inter-sample variance during individual trials and showing that the motor output samples demonstrate the synergic signature over time, V_UCM_ > V_ORT_, but only under continuous visual feedback. This inequality disappeared (V_UCM_ ≈ V_ORT_) when visual feedback was unavailable (see also De et al. 2024).

Note that the crucial importance of visual information for the control of force has been documented primarily during accurate force production in isometric conditions. Prehensile tasks involving object manipulation present a more complex picture, suggesting an interplay between sensory information of visual, haptic, and proprioceptive modalities. In particular, visual information may be unable to ensure proper feed-forward control of the moment of force applied to an asymmetrical object (Zhang et al. 2010; Craje et al. 2013; Shibata and Santello 2017). Under such conditions, even when the subjects observed the placement of an additional load or a change in the material density that changed the loading symmetry of the object, they were unable to avoid object tilt during the fast lifting action, although the tilt magnitude quickly reduced over a few trials. In other words, visual information was unable to ensure adequate time profiles of the total moment of force applied by the digits to the object, although visual information has been shown to be important for digit placement and swift digit-based force control (Bland et al. 2023). We would like to emphasize that in most of the cited studies deviations of performance variables from their desired values were quantified, i.e., the accuracy of performance of salient performance variables such as the vertical orientation of the object. We focus on the stability of performance variables, as reflected in the relative magnitudes of two variance components, V_UCM_ and V_ORT_. Since V_UCM_, by definition, has no effects on the salient performance variable, comparisons between such studies should be done with caution. The nature of the studied tasks could also play a role in the role of visual information: In the current study, isometric force production was studied, while in a number of cited earlier studies, the tasks involved object manipulation by the hand. Differences in the control of movement tasks and isometric force production tasks have been discussed recently (De et al. 2025).

During constant force production tasks, changing the frequency of presentation of visual feedback has a major impact on the accuracy of task performance (Sosnoff and Newell 2005, 2006). These observations are particularly relevant to our comparison of the continuous and off-target feedback conditions. In the off-target condition, force drifts could be expected, similar to those mentioned earlier. The size of the target naturally limited the magnitude of those drifts, but it still allowed larger force deviations from the center of the target, resulting in larger V_ORT_ (as observed in our study). Deviations along the UCM could also take place, but these were unlikely to be corrected since, by definition, they had no effect on the salient performance variable. Presenting continuous feedback also allowed the subjects to show variable accuracy of performance: Some of the subjects could be happy simply to keep the cursor inside the target, while others could strive towards perfection and tried to keep the cursor as close to the center of the target as possible during the trials. This could be the reason for the larger across-subjects variability in V_ORT_ under the continuous feedback compared to the off-target feedback, although the average magnitude of V_ORT_ was larger in the latter condition.

The control of force under the off-target visual feedback can be seen as consisting of two qualitatively different phases. When the cursor was inside the target, the feedback loop was ineffective, and force drifts similar to those described earlier could be expected over variable time intervals that took the cursor to reach the border of the target. When the cursor exited the target zone, visual feedback-based corrections were expected to bring the cursor back into the target. This type of control can be compared to the concept of intermittent control suggested for a range of actions from balancing an inverted pendulum with the hand to the control of posture during quiet standing (Bottaro et al. 2005; Loram et al. 2006; Gawthorpe et al. 2011).

It is of interest that the differences between the continuous and off-target feedback conditions were seen in all the variance variables, including V_TOT_, at the higher level of the hierarchy, i.e., when computed with respect to F_TOT_ as the sum of two F_HAND_ variables (Figs. 5, 6, and 7). These differences in V_TOT_ and V_UCM_ disappeared when analysis was performed at the lower level, i.e., when these indices were computed with respect to each F_HAND_ as the sum of the forces produced by the index and middle fingers of the corresponding hand. The effects were seen, however, for V_ORT_ reflecting the continuous vs. intermittent control. These findings emphasize the role of V_ORT_ modulation at the lower level as a factor defining the range of V_UCM_ at the higher level, as confirmed by the statistically significant correlations between these variables found in Fig. 7.

## Concluding comments

We would like to summarize the main findings from the described study. First, our results are the first to confirm experimentally a strong dependence between variance that does not affect performance (V_UCM_) at the top level of a hierarchy and variance that affects performance (V_ORT_) at the lower level. Moreover, this was confirmed across conditions associated with broadly varying performance-stabilizing synergy indices. Second, we have provided, so far, the most convincing dataset for the hierarchical control scheme with back-coupling feedback loops from sensory endings and within the CNS. Third, we have shown that analysis of inter-sample variance within individual trials is a feasible tool to explore one of the contributing factors to performance-stabilizing synergies, namely the one based on the back-coupling feedback loops. Combining inter-trial and intra-trial analysis of variance may provide an important toolbox to explore cases of impaired control of action stability in neurological patients (reviewed in Latash and Huang 2015).

The experiment suggests a few directions for future studies of action stability. For example, the role of intermittent visual feedback can be explored, possibly by varying the size of the targets in the off-target feedback condition. Processes within the UCM can be explored in more detail using analysis of long single trials. It seems feasible that these processes represent a superposition of random walk, drifts, and corrective actions (cf. Collins and DeLuca 1993, 1995; Loram and Lakie 2002; Loram et al. 2006), which have to be extracted and explored individually to understand their physiological origins and possible changes in cases of impaired control of action stability in clinical populations (reviewed in Latash and Huang 2015). In particular, such analysis can provide insight into the characteristic times of those processes, which may be expected to be longer within the UCM compared to the ORT (reflecting different stability of processes within the UCM and ORT).

Potentially, analysis of the two components of V_UCM_ can provide deeper insight into causes of the impaired stability of movements in people with neurological conditions (reviewed in Latash and Huang 2015; Vaz et al. 2019). Indeed, the low values of V_UCM_ in such patients can reflect lower V_UCM,SH_ and/or lower V_UCM,CoV_. If the former is true, one can conclude that these patients are impaired in the ability to map small changes in elemental variables on changes in the performance variables, i.e., in the ability to create the Jacobian of the system (cf. Goodman and Latash 2006). Such mapping is likely built on prior experience, and a smaller V_UCM,SH_ could imply a deficit in mechanisms of motor learning. Note that subcortical structures such as the basal ganglia and cerebellum have been strongly implicated in motor learning processes (reviewed in Shadmehr and Krakauer 2008), and impaired movement stability has been associated with deficits in transcortical loops involving those structures. This makes the hypothesis on lower V_UCM,SH_ highly feasible. On the other hand, if V_UCM,CoV_ is reduced, this implies an impaired ability to use sensory feedback and/or back-coupling loops within the CNS (cf. Martin et al. 2019) to facilitate co-variation of elemental variables stabilizing performance. The increased dependence of older persons and neurological patients on visual feedback and the effectiveness of augmented feedback (reviewed in Christou 2011; Kearney et al. 2019) make this scenario feasible as well. Quantitative studies of the two V_UCM_ components across clinical populations are needed to disambiguate the contributions of the components to the observed decrease in the V_UCM_. Such studies may have direct implications for the current and future treatment strategies.

It remains puzzling why somatosensory information from endings sensitive to force and deformation is apparently inadequate to provide functionally significant feedback for stabilization of finger force in isometric conditions leading to large force drifts in the absence of visual feedback (cf. Cuadra et al. 2021; De et al. 2024) while proprioception continues to be a reliable source of limb position information even after prolonged time without vision (Brown et al. 2003). This may be a reflection of yet unknown trade-offs between accurate perception of kinematic and kinetic variables that rely on information from the same groups of sensory endings estimated within the frameworks provided by the ongoing neural control process (reviewed in Feldman 2009; Latash 2018).

## Data Availability

Data are available by reasonable request to the corresponding author.

## List of symbols

*C*-command: Coactivation command
CNS: Central nervous system
F: Force
F_HAND_: Force produced by a hand
F^HAND^: A condition with visual feedback on force produced by a hand
F_IM_: Force produced by the index and middle fingers of a hand together
F_TOT_: Total force
F^TOT^: A condition with visual feedback on total force
FB_CONT_: Continuous visual feedback
FB_OFF-TARGET_: Visual feedback is shown only when the cursor is outside the target
IM_L_: The index and middle fingers of the left hand
IM_R_: The index and middle fingers of the right hand
J: Jacobian matrix
MU-mode: A group of motor units showing parallel changes in the firing frequency
MVC: Maximal voluntary contraction
ORT: Space orthogonal to the uncontrolled manifold
*R*-command: Reciprocal command
SD: Standard deviation
UCM: Uncontrolled manifold
V: Variance
V_ORT_: Variance along the ORT space
V_TOT_: Total variance
V_UCM_: Variance along the UCM
V_UCM,SH_: Contribution to V_UCM_ of variance in sharing a performance variable across elements
V_UCM,CoV_: Contribution to V_UCM_ of co-variation between element contributions over time
∆V: Index of performance-stabilizing synergy
∆V_Z_: Log-transformed values of ∆V
